# Sodium penta­fluoro­phenyl­borate

**DOI:** 10.1107/S1600536812042584

**Published:** 2012-10-20

**Authors:** Hannes Vitze, Hans-Wolfram Lerner, Michael Bolte

**Affiliations:** a Institut für Anorganische Chemie, J. W. Goethe-Universität Frankfurt, Max-von-Laue-Str. 7, 60438 Frankfurt/Main, Germany

## Abstract

The crystal structure of the title compound, Na[(C_6_F_5_)BH_3_], is composed of discrete anions and cations. The sodium cations are surrounded by four anions with three short Na⋯B [2.848 (8), 2.842 (7) and 2.868 (8) Å] and two short Na⋯F contacts [2.348 (5) and 2.392 (5) Å], forming a three-dimensional network. The anion is the first structural example of a pentafluorophenyl ring carrying a BH_3_ group.

## Related literature

For synthetic background, see: Schnurr *et al.* (2011 ▶). For a description of the Cambridge Structural Database, see: Allen (2002) ▶.
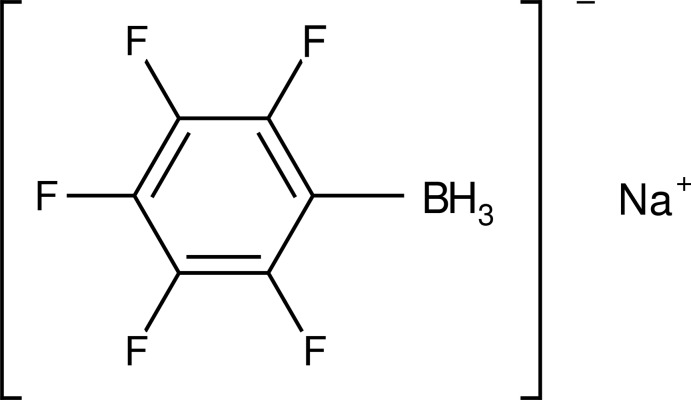


## Experimental

### 

#### Crystal data

Na^+^·C_6_H_3_BF_5_^−^
*M* *_r_* = 203.88
Monoclinic, 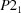

*a* = 4.6813 (10) Å
*b* = 6.1986 (16) Å
*c* = 12.993 (3) Å
β = 92.995 (17)°
*V* = 376.51 (15) Å^3^
*Z* = 2
Mo *K*α radiation
μ = 0.24 mm^−1^
*T* = 173 K
0.21 × 0.18 × 0.03 mm

#### Data collection

STOE IPDS II two-circle-diffractometer
Absorption correction: multi-scan (*MULABS*; Spek, 2009 ▶ and Blessing, 1995 ▶) *T*_min_ = 0.951, *T*_max_ = 0.993
2267 measured reflections
775 independent reflections
601 reflections with *I* > 2σ(*I*)
*R*_int_ = 0.116

#### Refinement

*R*[*F* ^2^ > 2σ(*F* ^2^)] = 0.058
*wR*(*F* ^2^) = 0.138
*S* = 1.01
775 reflections
119 parameters
1 restraint
H-atom parameters constrained
Δρ_max_ = 0.37 e Å^−3^
Δρ_min_ = −0.45 e Å^−3^

### 

Data collection: *X-AREA* (Stoe & Cie, 2001 ▶); cell refinement: *X-AREA*; data reduction: *X-AREA*; program(s) used to solve structure: *SHELXS97* (Sheldrick, 2008 ▶); program(s) used to refine structure: *SHELXL97* (Sheldrick, 2008 ▶); molecular graphics: *XP* in *SHELXTL* (Sheldrick, 2008 ▶); software used to prepare material for publication: *SHELXL97*.

## Supplementary Material

Crystal structure: contains datablock(s) I, global. DOI: 10.1107/S1600536812042584/ng5300sup1.cif

Structure factors: contains datablock(s) I. DOI: 10.1107/S1600536812042584/ng5300Isup2.hkl

Additional supplementary materials:  crystallographic information; 3D view; checkCIF report

## Figures and Tables

**Fig. 1.**
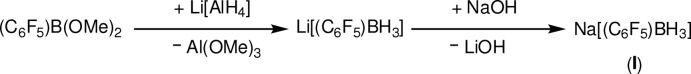
Reaction scheme for obtaining the title compound.

**Fig. 2.**
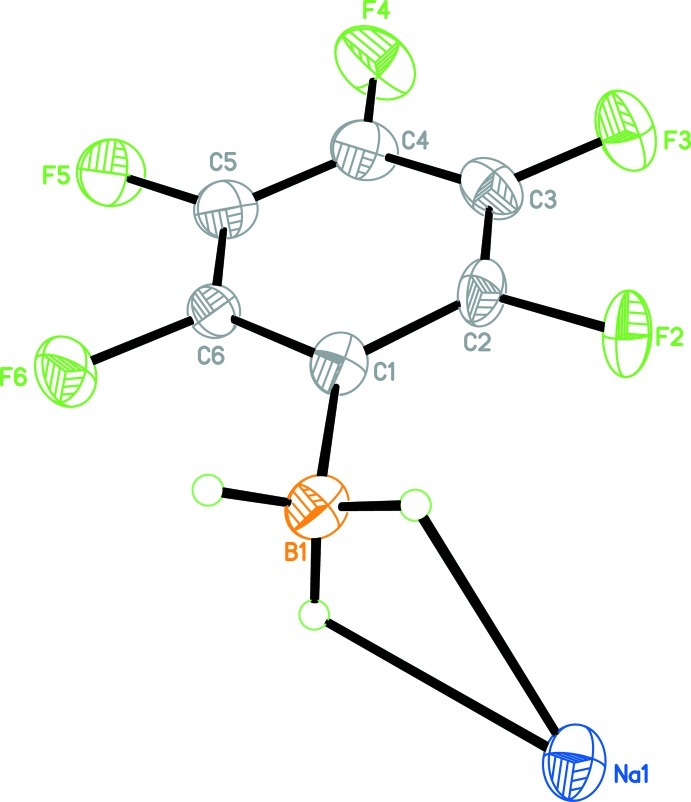
Perspective view of the title compound. Displacement ellipsoids are drawn at the 30% probability level.

**Fig. 3.**
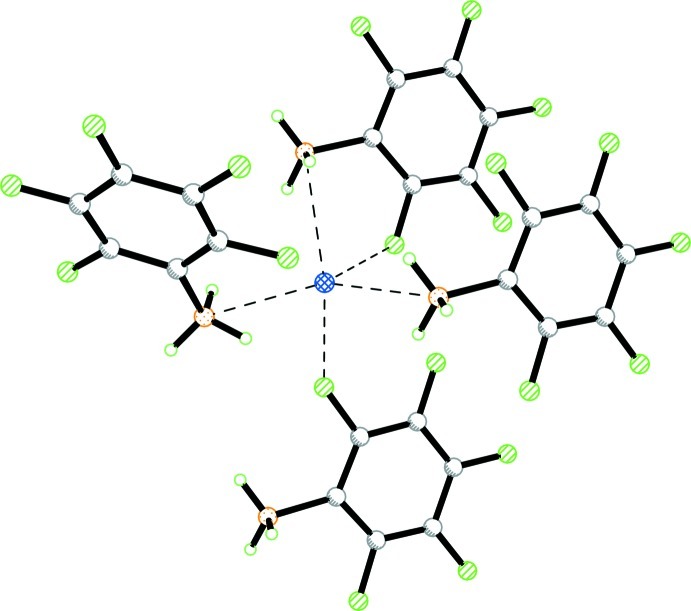
Environment of a sodium cation.

## supplementary crystallographic information

### Comment

The hydridoborates [(C_6_F_5_)BH_3_]^-^ and [(C_6_F_5_)_2_BH_2_]^-^ are convenient starting materials for the *in situ* generation of the boranes (C_6_F_5_)BH_2_ and (C_6_F_5_)_2_BH (Schnurr *et al.*, 2011). In this paper we report the crystal structure of Na[(C_6_F_5_)BH_3_] which was obtained from the reaction mixture of (C_6_F_5_)B(OMe)_2_ and Li[AlH_4_] by a cation exchange with NaOH (Fig. 1).

The title compound (Fig. 2) is composed of discrete anions and cations. The sodium cations are surrounded by four anions with three short Na···B [Na1···B1 2.848 (8) Å, Na1···B1^i^ 2.842 (7) Å, Na1···B1^ii^ 2.868 (8) Å; symmetry operators: (i) -*x* + 2, *y* - 1/2, -*z* + 1, (ii) -*x* + 1, *y* - 1/2, -*z* + 1] and two short Na···F [Na1···F6^ii^ 2.348 (5) Å, Na1···F2^ii^ 2.392 (5) Å] contacts (Fig. 3).

It is remarkable that this is the first structure with an pentafluorophenyl ring carrying a BH_3_ group. A search in the Cambridge Crystallographic Database (CSD, Version 5.33 of November 2011, plus three updates (Allen, (2002). *Acta Cryst.* B58, 380–388) yielded no hit at all for this fragment.

### Experimental

In a round bottom flask (C_6_F_5_)B(OMe)_2_ (0.16 g, 0.65 mmol) was dissolved in 10 ml diethyl ether. Under stirring a 1 m solution of Li[AlH_4_] in diethyl ether (1.2 mmol, 1.1 ml) was added *via* canula. A brown slurry was obtained which was treated with 3 ml aqueous NaOH (3 mmol) and 15 ml benzene. Insoluble material was removed by filtration from the organic layer. Single crystals of the title compound were obtained of the concentrated benzene solution (5 ml). Yield 20%.

### Refinement

Due to the absence of anomalous scatterers, the absolute structure could not be determined and 414 Friedel pairs were merged. H atoms were located in a difference map, but geometrically positioned and refined using a riding model with fixed individual displacement parameters [U(H) = 1.5 *U*_eq_(B)] and with B—H = 0.98 Å. The BH_3_ group was allowed to rotate but not to tip.

### Crystal data

**Table:**

Na^+^·C_6_H_3_BF_5_^−^	*F*(000) = 200
*M**_r_* = 203.88	*D*_x_ = 1.798 Mg m^−^^3^
Monoclinic, *P*2_1_	Mo *K*α radiation, λ = 0.71073 Å
Hall symbol: P 2yb	Cell parameters from 1888 reflections
*a* = 4.6813 (10) Å	θ = 3.7–25.5°
*b* = 6.1986 (16) Å	µ = 0.24 mm^−^^1^
*c* = 12.993 (3) Å	*T* = 173 K
β = 92.995 (17)°	Plate, colourless
*V* = 376.51 (15) Å^3^	0.21 × 0.18 × 0.03 mm
*Z* = 2	

### Data collection

**Table:**

STOE IPDS II two-circle-diffractometer	775 independent reflections
Radiation source: fine-focus sealed tube	601 reflections with *I* > 2σ(*I*)
Graphite monochromator	*R*_int_ = 0.116
ω scans	θ_max_ = 25.6°, θ_min_ = 3.6°
Absorption correction: multi-scan (*MULABS*; Spek, 2009 and Blessing, 1995)	*h* = −5→5
*T*_min_ = 0.951, *T*_max_ = 0.993	*k* = −6→7
2267 measured reflections	*l* = −15→15

### Refinement

**Table:**

Refinement on *F*^2^	Primary atom site location: structure-invariant direct methods
Least-squares matrix: full	Secondary atom site location: difference Fourier map
*R*[*F*^2^ > 2σ(*F*^2^)] = 0.058	Hydrogen site location: inferred from neighbouring sites
*wR*(*F*^2^) = 0.138	H-atom parameters constrained
*S* = 1.01	*w* = 1/[σ^2^(*F*_o_^2^) + (0.0711*P*)^2^] where *P* = (*F*_o_^2^ + 2*F*_c_^2^)/3
775 reflections	(Δ/σ)_max_ < 0.001
119 parameters	Δρ_max_ = 0.37 e Å^−^^3^
1 restraint	Δρ_min_ = −0.45 e Å^−^^3^

### Special details

**Table:**

Experimental. ;
Geometry. All e.s.d.'s (except the e.s.d. in the dihedral angle between two l.s. planes) are estimated using the full covariance matrix. The cell e.s.d.'s are taken into account individually in the estimation of e.s.d.'s in distances, angles and torsion angles; correlations between e.s.d.'s in cell parameters are only used when they are defined by crystal symmetry. An approximate (isotropic) treatment of cell e.s.d.'s is used for estimating e.s.d.'s involving l.s. planes.
Refinement. Refinement of *F*^2^ against ALL reflections. The weighted *R*-factor *wR* and goodness of fit *S* are based on *F*^2^, conventional *R*-factors *R* are based on *F*, with *F* set to zero for negative *F*^2^. The threshold expression of *F*^2^ > σ(*F*^2^) is used only for calculating *R*-factors(gt) *etc*. and is not relevant to the choice of reflections for refinement. *R*-factors based on *F*^2^ are statistically about twice as large as those based on *F*, and *R*- factors based on ALL data will be even larger.

### Fractional atomic coordinates and isotropic or equivalent isotropic displacement parameters (Å^2^)

**Table:**

	*x*	*y*	*z*	*U*_iso_*/*U*_eq_	
Na1	0.8015 (5)	0.5576 (5)	0.5911 (2)	0.0289 (6)	
B1	0.7013 (14)	0.7940 (13)	0.4048 (5)	0.0245 (15)	
H1A	0.6928	0.9477	0.3873	0.037*	
H1B	0.8948	0.7573	0.4316	0.037*	
H1C	0.5643	0.7628	0.4573	0.037*	
C1	0.6218 (11)	0.6519 (12)	0.3025 (5)	0.0228 (13)	
C2	0.7487 (11)	0.4581 (11)	0.2778 (5)	0.0258 (14)	
C3	0.6739 (13)	0.3361 (12)	0.1908 (5)	0.0297 (15)	
C4	0.4582 (12)	0.4078 (14)	0.1233 (5)	0.0325 (16)	
C5	0.3237 (12)	0.6030 (13)	0.1438 (5)	0.0316 (18)	
C6	0.4083 (12)	0.7149 (12)	0.2310 (5)	0.0275 (15)	
F2	0.9668 (7)	0.3764 (7)	0.3429 (3)	0.0351 (10)	
F3	0.8099 (8)	0.1482 (7)	0.1730 (3)	0.0420 (11)	
F4	0.3794 (9)	0.2917 (10)	0.0395 (3)	0.0495 (13)	
F5	0.1147 (7)	0.6743 (8)	0.0784 (3)	0.0426 (12)	
F6	0.2667 (7)	0.9043 (7)	0.2468 (3)	0.0351 (10)	

### Atomic displacement parameters (Å^2^)

**Table:**

	*U*^11^	*U*^22^	*U*^33^	*U*^12^	*U*^13^	*U*^23^
Na1	0.0223 (10)	0.0281 (14)	0.0361 (14)	−0.0006 (11)	−0.0003 (9)	0.0047 (13)
B1	0.020 (3)	0.028 (4)	0.026 (3)	−0.002 (3)	0.000 (3)	0.002 (4)
C1	0.015 (2)	0.025 (3)	0.028 (3)	−0.005 (2)	0.001 (2)	0.004 (3)
C2	0.017 (2)	0.028 (3)	0.032 (4)	0.003 (3)	−0.005 (2)	0.007 (3)
C3	0.028 (3)	0.025 (4)	0.037 (4)	0.001 (3)	0.008 (3)	−0.006 (3)
C4	0.023 (3)	0.044 (4)	0.029 (3)	−0.002 (3)	−0.002 (3)	−0.006 (3)
C5	0.018 (2)	0.047 (5)	0.028 (4)	0.001 (3)	−0.002 (2)	0.003 (3)
C6	0.018 (3)	0.039 (4)	0.027 (3)	0.007 (3)	0.007 (2)	0.007 (3)
F2	0.0276 (18)	0.031 (2)	0.045 (2)	0.0079 (18)	−0.0124 (16)	0.003 (2)
F3	0.040 (2)	0.033 (2)	0.052 (3)	0.012 (2)	0.0000 (18)	−0.009 (2)
F4	0.039 (2)	0.066 (3)	0.043 (3)	0.006 (2)	−0.0056 (18)	−0.022 (3)
F5	0.0262 (17)	0.068 (3)	0.033 (2)	0.015 (2)	−0.0083 (15)	0.005 (2)
F6	0.0262 (17)	0.044 (3)	0.035 (2)	0.0156 (18)	−0.0050 (15)	−0.002 (2)

### Geometric parameters (Å, º)

**Table:**

Na1—F6^i^	2.348 (5)	C2—C3	1.389 (10)
Na1—F2^ii^	2.392 (5)	C3—F3	1.353 (9)
Na1—B1^iii^	2.842 (7)	C3—C4	1.376 (10)
Na1—B1^i^	2.868 (8)	C4—F4	1.342 (9)
B1—C1	1.621 (10)	C4—C5	1.396 (11)
B1—H1A	0.9800	C5—F5	1.337 (7)
B1—H1B	0.9800	C5—C6	1.370 (10)
B1—H1C	0.9800	C6—F6	1.369 (8)
C1—C6	1.385 (9)	F2—Na1^iii^	2.392 (5)
C1—C2	1.385 (10)	F6—Na1^iv^	2.348 (5)
C2—F2	1.387 (7)		
			
F6^i^—Na1—F2^ii^	95.35 (17)	F2—C2—C1	119.1 (6)
F6^i^—Na1—B1^iii^	84.3 (2)	C3—C2—C1	124.6 (6)
F2^ii^—Na1—B1^iii^	96.5 (2)	F3—C3—C4	120.3 (7)
F6^i^—Na1—B1^i^	66.57 (18)	F3—C3—C2	120.5 (6)
F2^ii^—Na1—B1^i^	145.2 (2)	C4—C3—C2	119.2 (6)
B1^iii^—Na1—B1^i^	110.1 (3)	F4—C4—C3	120.4 (7)
C1—B1—H1A	109.5	F4—C4—C5	120.8 (6)
C1—B1—H1B	109.5	C3—C4—C5	118.8 (6)
H1A—B1—H1B	109.5	F5—C5—C6	121.9 (7)
C1—B1—H1C	109.5	F5—C5—C4	119.2 (6)
H1A—B1—H1C	109.5	C6—C5—C4	118.9 (6)
H1B—B1—H1C	109.5	F6—C6—C5	115.9 (6)
C6—C1—C2	113.1 (6)	F6—C6—C1	118.6 (6)
C6—C1—B1	121.6 (6)	C5—C6—C1	125.4 (6)
C2—C1—B1	125.3 (6)	C2—F2—Na1^iii^	145.7 (4)
F2—C2—C3	116.3 (6)	C6—F6—Na1^iv^	124.9 (4)
			
C6—C1—C2—F2	179.8 (5)	F4—C4—C5—C6	179.2 (6)
B1—C1—C2—F2	−1.5 (8)	C3—C4—C5—C6	−0.9 (9)
C6—C1—C2—C3	−0.1 (8)	F5—C5—C6—F6	−0.3 (9)
B1—C1—C2—C3	178.6 (6)	C4—C5—C6—F6	−179.6 (6)
F2—C2—C3—F3	0.2 (9)	F5—C5—C6—C1	179.8 (6)
C1—C2—C3—F3	−179.9 (6)	C4—C5—C6—C1	0.5 (9)
F2—C2—C3—C4	179.7 (6)	C2—C1—C6—F6	−179.9 (5)
C1—C2—C3—C4	−0.4 (9)	B1—C1—C6—F6	1.4 (8)
F3—C3—C4—F4	0.4 (10)	C2—C1—C6—C5	0.0 (9)
C2—C3—C4—F4	−179.2 (6)	B1—C1—C6—C5	−178.7 (6)
F3—C3—C4—C5	−179.6 (6)	C3—C2—F2—Na1^iii^	−30.8 (9)
C2—C3—C4—C5	0.9 (9)	C1—C2—F2—Na1^iii^	149.3 (5)
F4—C4—C5—F5	−0.2 (9)	C5—C6—F6—Na1^iv^	151.8 (4)
C3—C4—C5—F5	179.7 (6)	C1—C6—F6—Na1^iv^	−28.2 (7)

Symmetry codes: (i) −*x*+1, *y*−1/2, −*z*+1; (ii) −*x*+2, *y*+1/2, −*z*+1; (iii) −*x*+2, *y*−1/2, −*z*+1; (iv) −*x*+1, *y*+1/2, −*z*+1.
